# Intracardiac Atrial Signal Amplitude In Congenital And Acquired Complete Heart Block

**Published:** 2010-10-31

**Authors:** Mohammad Reza Samiei Nasab, Mohammad Reza Dehghani, Mehrdad Taherioun, Alireza Rostamzadeh

**Affiliations:** 1 Department of Electrophysiology, Chamran Heart Hospital, Isfahan University of Medical Science, Isfahan, Iran; 2 Department of Electrophysiology, Seyyed-O-Shohada Heart Hospital, Uromieh University of Medical Science, Uromieh, Iran

**Keywords:** Atrial signal amplitude, Congenital CHB, VDD pacemaker

## Abstract

**Background:**

Good and reliable atrial sensing is a fundamental part of atrioventricular (AV) synchrony in dual chamber pacemakers. Due to the floating nature of atrial sensing electrode in single pass dual chamber pacemakers (VDD) compared with two-lead dual chamber pacemakers (DDD), they are more prone to atrial under-sensing and the resulting loss of AV synchrony. We hypothesized that there is a relation between the chronicity of AV block and the amplitude of intracardiac atrial signal amplitudes (IASA).

**Methods:**

Detailed demographic, electrocardiographic and echocardiographic data were recorded in 34 consecutive patients with congenital and acquired complete heart block (CHB). The intracardiac atrial signal amplitudes (IASA) were recorded at implantation time, 48 hours and 2 months post-implantation and compared between the two groups of patients.

**Results:**

The mean age of the study group was 38.73±12.53 years (congenital: 30.08±11.07, acquired: 47.38±6.5). There were no important differences in left atrial or ventricular sizes and in P-wave amplitude in lead II, but the IASA was significantly higher in the congenital group at implantation time (5.21±1.86 vs. 3.38±0.84 mV, P<0.001) and during the follow-up.

**Conclusion:**

The intracardiac atrial signal amplitudes were higher in congenital CHB compared with the acquired CHB. Chronicity (and may be the congenital type) of CHB may be an affecting factor in case selection for VDD pacemaker implantation.

## Introduction

With maintenance of AV synchrony in dual chamber pacemakers (compared to ventricular pacing only), patients may experience better hemodynamics and improved exercise tolerance. Furthermore, they may have a lower chance for developing atrial fibrillation (AF), heart failure and cerebral embolism in the future [1]. One of the main factors in maintenance of AV synchrony is good atrial sensing.

It's been more than three decades since single pass VDD pacemakers were introduced [2]. Despite their suggested advantages such as lower costs, less implantation times, fewer complications and also lower potential for development of AF, they are still underutilized. This underutilization is attributed to the fear of later on development of sinus node dysfunction or atrial undersensing [3-6].

Hitherto, the effects of factors such as breathing, body position and exercise in atrial undersensing have been discussed in various investigations  [7-10]. In general, there is a tendency towards decreasing atrial signal amplitude during the first several days to weeks after lead implantation due to inflammatory response and edema at the electrode tissue interface [11]. Moreover, a remarkable difference has been reported between signal amplitude measured by an analyzer at the time of implantation, and the one measured during the device interrogation, with the signal amplitude being lower in the latter one [6]. Therefore, it would be logical to assume that the more the signal amplitude at the time of implantation, the less the possibility of later development of undersensing.

In order to overcome the latter problem, many suggestions have been made such as using active autosensing algorithms, right sided implantation, and programming to the highest atrial sensitivity. Nevertheless intermittent atrial undersensing still remains as a problem [12-14].

Burri et al. discovered that there is a direct correlation between the maximal surface P-wave amplitude and intra atrial signal amplitude (IASA) measured with a free-floating atrial dipole in VDD pacemakers. As well, Wiegand et al. and Brandt et al. have separately suggested the existence of a reverse correlation between the age and IASA in VDD and DDD pacemakers respectively [4,15-17].

There are no data about the relation between IASA and the chronicity of AVB so far. In this small-scale study we tried to compare patients with congenital and acquired AV block to test the hypothesis that there is a correlation between the duration of AV block and the IASA.

## Patients and Methods

We analyzed the data from 17 patients with congenital CHB (age>18 years) and 17 patients with acquired CHB (age<60 years) who received two-lead DDD pacemakers at a referral hospital between September 2005 and December 2009. The indication for pacemaker implantation was symptomatic CHB. All patients with a history of open heart surgery were excluded to avoid the need for active fixation atrial lead. Written informed consents were obtained from all patients and the study was approved by local Ethics committee, and. The mean age at implantation was 38.73±12.53 years, and 62% of the patients were women (82% in congenital group versus 42% in acquired group). In the acquired group the underlying diseases were hypertension (30%), ischemic heart disease (23%), and myocarditis (7.7%). The pacing system in both groups included: Medtronic Kappa DR 901 generators (Minneapolis, MN, USA), Medtronic Capsure sense 4574-53passive fixation atrial leads, and Medtronic Capsure fix 4076-58 active fixation ventricular leads.

### Implantation Technique

Devices were implanted in electrophysiology laboratory using standard implantation techniques by a qualified electrophysiologist. All the leads were implanted through left subclavian vein using 7 French peel-away sheaths. The active fixation ventricular leads were implanted in right ventricular outflow tract (RVOT) or high right ventricular septum. All passive fixation atrial leads were placed in right atrial (RA) appendage. Atrial and ventricular sensing and pacing thresholds and lead impedances were measured by pacing system analyzer (Medtronic Inc., Model: 2090EN). The acceptable atrial pacing and sensing threshold measured by the analyzer were <1.0V and ≥ 1.5 mV, respectively. After the leads were secured in the pocket, they were anchored to DDD pulse generator.

### Follow Up

Atrial signal amplitudes were measured in supine position at predischarge and two months after implantation with the same programmer (Medtronic Inc. Model: 2090EN).

### Definitions

Congenital CHB was diagnosed by reviewing the previous surface electrocardiograms and medical files of the patients in the absence of new events (by history and physical examination).

### Analysis of Data

Continuous variables are displayed as mean ±SD and qualitative variables as numbers. Student t-test, Mann-Whitney U-test, and chi-square (Fisher exact) test were used for univariate comparison of parameters. A P-value of ≤ 0.05 was considered statistically significant. GLM was used for age and sex adjustment.

## Results

34 patients having symptomatic CHB (17 patients with congenital and 17 patients with acquired CHB) were entered into our study. The clinical and paraclinical characteristics of the patients are presented in Table 1. Surprisingly, more than 80% of the patients in the congenital group were women (p=0.015). The patients in the congenital group were younger than the acquired group (30.08±11.07 vs. 47.38± 6.50 years, P<0.001).

**Figure 1:**
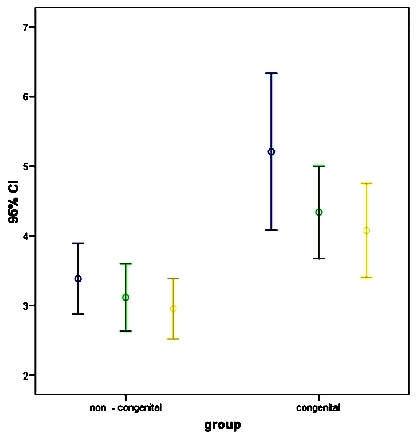

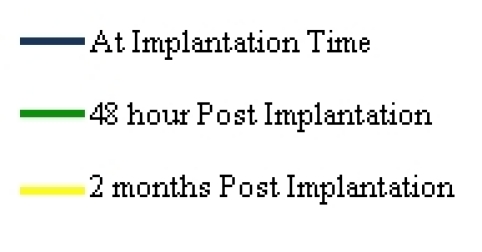
Comparison of IASA at implantation time and during follow up in both group.

There were no significant differences in body mass index, and left atrial (LA) or right ventricular (RV) sizes in echocardiography. Five patients (38.5%) in the acquired group had RV dysfunction. Low left ventricular (LV) ejection fraction was more common in the acquired group (45.77% ±5.34 vs. 55.38% ±11.63, P=0.015). The right atrial (RA) size was higher in the acquired group (3.60 cm ±0.5 vs. 3.22 cm ±o.28, P=0.023). P-wave duration in lead II (2.37mm ±0.36) and amplitude in lead V1 (1.54 mV ±0.43) were significantly higher in the congenital group (p=0.006, P=0.034, respectively). IASA levels were significantly higher in the congenital group at implantation time (5.21±1.86 vs. 3.38±0.84 mm, P<0.001) and remained higher during the follow up. (Table 1) (Figure 1).

## Discussion

The results of the present study indicate higher IASA levels in patients with congenital CHB. Studying the fetuses with congenital CHB, Li et al. concluded that these fetuses have surface P waves with higher amplitude and longer duration. He suggested that the hypertrophy of the atrial myocytes, as a compensatory mechanism in response to low heart rates in these fetuses, was the responsible for these findings one [18]. In heart blocks with the ensuing AV dyssynchrony (AVD), atria could undergo hypertrophic changes. Considering the fact that in patients with congenital CHB, AVD usually lasts longer than in patients with acquired CHB; more hypertrophy would be expected in the former cases. In our study too, the amplitude and duration of surface P waves were longer in patients with congenital CHB although this difference was statistically considerable only in terms of duration.

The cause of the reverse correlation between age and IASA level were suggested to lay in the electrophysiologic changes of atrial cells due to the increasing age one [17]. Although in our study we tried to choose two groups of patients with the least age differences, this difference was still statistically significant. However, after adjusting for age, the IASA differences between the two groups persisted. As patients presenting with symptomatic acquired CHB can be assumed to have a recent onset of disease, the chronicity of development of CHB may be suggested to account for the differences in IASA levels in the two groups of patients.

Structural heart diseases are less common in younger patients and the sizes of the atria are smaller. These factors may also be the reason for higher IASA levels in younger patients. In this study, the right atrial sizes in the acquired group were larger than that of the congenital ones (P=0.023) but it was not found to be significant once analyzed by Pearson correlation test.

Osadchii  et al. explained how myocardial cell hypertrophy occurs much easier in response to adrenergic over-activation one [19]. The reason may be the longer contact of the congenital CHB group with circulatory catecholamines that facilitate atrial hypertrophy in these patients compared with the acquired group.

## Limitations

This study had its own limitations. This study failed to measure circulatory catecholamine levels and compares them in two groups. It might have been helpful if we performed trans-esophageal echocardiography (TEE) to measure the thickness of the atrial walls, and the amount of their hypertrophy. Our small sample size would not allow for defining age quartiles in the patients with congenital CHB. This would allow for further investigation of the role of chronicity on IASA levels. A larger study would be more revealing.

## Conclusion

This study suggests that patients suffering from congenital CHB have higher IASA levels compared with the acquired CHB patients. The chronicity of CHB may be the primary factor in determining the IASA levels. These patients may be better candidates to receive VDD pacemakers and less prone to the development of atrial undersensing in the future.

## Figures and Tables

**Table 1:**
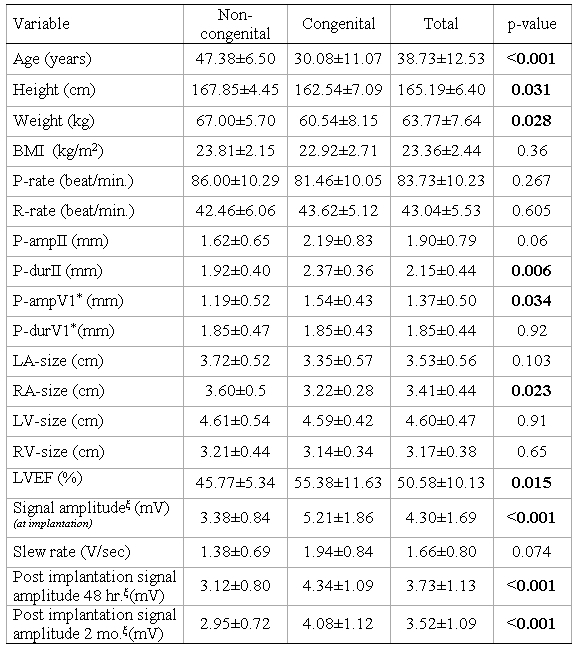
Clinical characteristics of subjects according to group

Data are expressed in mean ± SD P-value: obtained from t-test.  Significant level P-value=0.05 * P-value: obtained from Mann-Whitney (because of non-normality) ξ  P-value: obtained from univariate analyze (adjusted by age and sex)
